# Intraoperative Margin Control in Eyelid Tumor Surgery: Current Standards, Imaging Advances, and Emerging Techniques

**DOI:** 10.3390/curroncol33050273

**Published:** 2026-05-08

**Authors:** Michele Nardella, Anna Argentesi, Claudia Pirro, Claudia Quaranta Leoni, Francesco M. Quaranta Leoni

**Affiliations:** 1 Department of Translational Medicine, University of Ferrara, 44121 Ferrara, Italy; nrdmhl@unife.it (M.N.); rgnnna@unife.it (A.A.); 2 Department of Ophthalmology, Ospedali Privati Forlì “Villa Igea”, 47122 Forlì, Italy; 3 Oftalmoplastica Roma, 00197 Roma, Italy; claudiapirro@icloud.com; 4 Catholic University of the Sacred Heart, 00168 Roma, Italy; claudia.quarantaleoni01@icatt.it; 5 Orbital and Adnexal Service, Tiberia Hospital—GVM Care & Research, 00137 Roma, Italy

**Keywords:** eyelid tumors, periocular oncology, Mohs micrographic surgery, frozen section, intraoperative margin control, confocal microscopy, optical coherence tomography, photoacoustic imaging, artificial intelligence

## Abstract

**Background:** Eyelid malignancies require accurate intraoperative margin control to achieve complete tumor excision while preserving the functional and aesthetic integrity of the periocular region. Mohs micrographic surgery (MMS) is widely regarded as the reference standard for margin-controlled excision, whereas frozen section–controlled excision (FSC) represents a reliable and widely used alternative in oculoplastic practice. In parallel, several emerging imaging technologies are being investigated to improve real-time tumor detection and surgical precision. **Methods:** A narrative review of the literature was conducted to summarize current evidence on intraoperative margin control in eyelid tumor surgery. The review focused on established surgical techniques, including MMS and FSC, as well as emerging imaging modalities such as fluorescence confocal microscopy, reflectance confocal microscopy, optical coherence tomography, line-field confocal optical coherence tomography, photoacoustic imaging, and artificial intelligence (AI)-assisted analysis. **Results:** MMS provides complete circumferential peripheral and deep margin assessment and remains the benchmark for high-risk, recurrent, and poorly defined periocular tumors, particularly basal cell carcinoma. FSC offers favorable oncologic outcomes, allows immediate reconstruction, and remains an effective option when MMS is not available. Emerging imaging modalities have shown promising diagnostic performance for tumor detection, presurgical mapping, and intraoperative support, particularly in basal cell carcinoma, although evidence in periocular tumors remains limited for most techniques. AI-assisted approaches have also demonstrated high accuracy in the interpretation of frozen sections and optical imaging data, suggesting potential to improve workflow efficiency and diagnostic consistency. **Conclusions:** MMS and FSC remain the current standards for intraoperative margin control in eyelid tumor surgery. Emerging imaging technologies and AI-based tools may further enhance surgical precision and tissue preservation, but most remain investigational in the periocular setting. Further prospective studies are needed to validate their clinical utility, define standardized workflows, and clarify their role alongside established histopathologic techniques.

## Simple Summary

Eyelid tumors require highly precise surgical management because the eyelids play a critical role in protecting the ocular surface, and their delicate structures must be preserved whenever possible. The primary challenge is to achieve complete tumor removal while minimizing injury to surrounding healthy tissue. Specialized intraoperative techniques enable surgeons to evaluate surgical margins in real time and confirm that no malignant cells remain. Among these methods, Mohs micrographic surgery is widely considered the gold standard because of its high cure rates and maximal tissue conservation. Frozen section-controlled excision is another suitable approach, particularly in settings where Mohs surgery is not available. Emerging imaging technologies, including confocal microscopy, optical coherence tomography, photoacoustic imaging, and artificial intelligence–assisted analysis, may further enhance surgical precision and efficiency. Over time, these advances could broaden access to high-quality care, lower recurrence rates, and support more personalized treatment strategies.

## 1. Introduction

Eyelid malignancies account for approximately 5–10% of all cutaneous cancers and present unique surgical challenges due to the anatomical complexity and functional importance of the periocular region. The eyelids play a critical role in protecting the ocular surface, maintaining tear film stability, and preserving visual function [1]. Complete surgical excision with histologically clear margins is therefore essential to minimize recurrence while preserving eyelid integrity and allowing optimal functional and aesthetic reconstruction [2].

Basal cell carcinoma (BCC) accounts for ~90% of eyelid malignancies in Caucasian populations, followed by squamous cell carcinoma (SCC). Sebaceous gland carcinoma (SGC), malignant melanoma (MM), and Merkel cell carcinoma (MCC) are less frequent but clinically important due to aggressive behavior and diagnostic complexity [3]. The prevalence of eyelid malignancies varies significantly by ethnicity; in Asian populations, SGC represents one of the most common malignant eyelid tumors [4].

Regardless of tumor type, periocular malignancies frequently exhibit subclinical extension and irregular growth patterns, particularly in aggressive histologic variants, recurrent tumors, and lesions located in high-risk areas such as the medial canthus. These characteristics make accurate intraoperative assessment of tumor margins a crucial component of surgical management [2,4].

Two principal techniques are currently used to achieve intraoperative margin control in eyelid tumor surgery: Mohs micrographic surgery (MMS) and frozen section–controlled excision (FSC) [5,6]. MMS provides complete circumferential and deep margin analysis using en face sectioning, allowing precise identification of residual tumor while maximizing tissue conservation [6,7,8]. FSC, widely used in oculoplastic surgery, offers an alternative strategy that combines intraoperative histopathologic evaluation with single-stage tumor excision and reconstruction [9].

Technological advances are expanding the possibilities for real-time intraoperative tumor assessment. Emerging modalities—including fluorescence confocal microscopy (FCM), reflectance confocal microscopy (RCM), optical coherence tomography (OCT), Line-field Confocal OCT (LC-OCT), photoacoustic imaging (PI), and AI–assisted analysis—are enhancing intraoperative precision and may complement traditional histopathologic techniques in the future [10,11,12,13,14,15].

This review summarizes current standards and emerging technologies for margin control in eyelid tumor surgery, emphasizing their role in improving oncologic and functional outcomes.

## 2. Materials and Methods

A narrative review methodology was employed to synthesize current evidence on intraoperative margin control in eyelid tumor surgery, with particular focus on MMS, FSC, and emerging imaging modalities that support real-time surgical decision-making.

A comprehensive literature search was conducted using PubMed/MEDLINE, Embase, and Google Scholar databases to identify relevant studies published between January 1990 and March 2026. The search strategy combined Medical Subject Headings (MeSH) terms and free-text keywords related to periocular malignancies and intraoperative margin assessment. Representative search strategies included combinations of terms such as “eyelid tumors” or “periocular malignancies” with “Mohs surgery,” “micrographic surgery,” “frozen section,” or “margin control”; tumor-specific terms including “basal cell carcinoma,” “squamous cell carcinoma,” “sebaceous carcinoma,” "Merkel cell carcinoma", and “melanoma” combined with “eyelid” or “periocular” and “surgical margins” or “intraoperative assessment”; and emerging technology–related terms such as “confocal microscopy,” “optical coherence tomography,” “photoacoustic imaging,” and “artificial intelligence” combined with “skin cancer” or “eyelid tumors.” The search was supplemented by manual screening of reference lists from relevant articles and review papers.

Titles and abstracts were independently screened by the authors to identify studies relevant to intraoperative margin control in eyelid tumor surgery, and full-text articles were subsequently assessed for eligibility. Studies were considered eligible if they addressed surgical margin control techniques, including MMS, FSC, or paraffin-based approaches, reported clinically relevant outcomes such as recurrence rates or margin status, evaluated imaging modalities applicable to periocular tumors, or provided meaningful clinical insights into periocular malignancy management. Studies were excluded if they were not directly related to margin assessment, included only experimental or laboratory data without clinical application, consisted solely of isolated case reports without broader clinical relevance, lacked an accessible English abstract, or represented duplicate publications.

**Figure 1:**
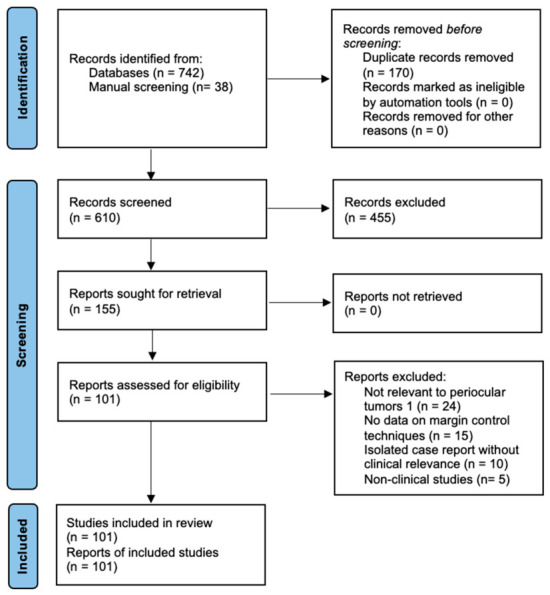
PRISMA 2020 flow diagram of the study selection process.

The literature search identified 742 records through database searching and 38 additional records through manual screening. After removal of duplicates, 610 records were screened based on title and abstract, of which 155 articles underwent full-text review. Following eligibility assessment, a total of 101 studies were included in the qualitative synthesis. The study selection process is summarized in a PRISMA 2020 flow diagram (Figure 1).

For each included study, relevant data were extracted, including tumor type, patient population, surgical technique, margin assessment method, recurrence outcomes, diagnostic performance where applicable, and practical considerations such as workflow, availability, and limitations. Given the heterogeneity in study designs, populations, and reported outcomes, findings were synthesized qualitatively using a narrative approach rather than formal meta-analysis.

## 3. Results

### 3.1. Overview of Eyelid Malignancies

A wide spectrum of malignant tumors can arise in the periocular region from the epidermis, dermis, or adnexal structures of the eyelid [16].

Periocular BCC typically appears as a pearly, erythematous papule with visible telangiectasia and, in some cases, central ulceration. However, its presentation can vary and may include a thin, scaly plaque, a persistent ulcer, or localized loss of eyelashes, particularly when the tumor involves the eyelid margin. The lower eyelid is the most frequently affected site, accounting for ~50% of periorbital BCCs. This predilection is likely due to its greater exposure to ultraviolet radiation [17]. The medial canthus is the second most common location, involved in 17–29% of cases. This site is considered high-risk, as it overlies an embryologic cleavage plane that allows tumors to more easily invade deeper anatomical structures, including the lacrimal system, periosteum, posterior orbit, paranasal sinuses, anterior cranial fossa, and even the nasopharynx [18]. Histologic subtype also plays a critical role in determining tumor biological behavior. Nodular BCC is the most prevalent subtype in the periocular region, accounting for approximately 54% of cases. Other variants include infiltrative (15%), superficial (9.7%), micronodular (5.7%), morpheaform or sclerosing (4.2%), basosquamous (1.9%), adenoid (1.8%), and pigmented (0.7%). Among these, infiltrative, morpheaform, and basosquamous subtypes are considered more aggressive, with a greater propensity for subclinical extension and invasion of adjacent structures [16,18]. Perineural invasion (PNI) represents an important adverse prognostic factor. In a multicenter series, periocular BCC with PNI demonstrated a five-year recurrence rate of approximately 7.7% after treatment [18]. The treatment of choice for periocular BCCs is surgical excision of the lesion with margin control. BCC carries an excellent prognosis when surgical margins are histologically clear [17].

SCC accounts for approximately 5–10% of eyelid malignancies and exhibits a higher potential for local invasion and metastasis compared with BCC [19], with risk factors including UV exposure, immunosuppression, and chronic skin lesions; eyelid SCC may also arise from conjunctival ocular surface squamous neoplasia (OSSN) extension. Poor differentiation and PNI are adverse prognostic factors associated with higher recurrence rates, particularly in advanced T stages. Diagnosis may be challenging in poorly differentiated cases, where immunohistochemical markers (e.g., androgen receptor and adipophilin) help distinguish it from SGC [20]. Staging influences management and prognosis; tumors ≥ T2b require a systemic work-up with imaging such as head and neck ultrasound, magnetic resonance imaging (MRI), and PET/CT. Treatment typically involves surgical excision with margin control, with adjuvant radiotherapy indicated in cases of PNI or vascular invasion. Adjunctive treatments include topical therapies for associated OSSN (e.g., interferon, 5-FU, Mitomycin C), while systemic agents such as acitretin and PD-1 inhibitors may have a role in advanced or neoadjuvant settings [21,22].

SGC is a rare but aggressive eyelid tumor, most often arising from meibomian glands of the upper eyelid in elderly patients, particularly women. It may present as in situ or invasive disease and frequently mimics benign conditions such as blepharitis or chalazion; pagetoid spread across the conjunctiva is common and can delay diagnosis [23]. Diagnosis requires full-thickness eyelid surgical excision and conjunctival mapping biopsies, with evaluation for mismatch repair defects and possible association with Muir-Torre syndrome. Imaging is essential for staging, especially in tumors > 20 mm. Whole-body PET/CT may be used to detect regional or distant metastases. PET/CT is ideally performed before surgery to avoid false positives from postoperative inflammation and is often repeated at 3 months as a baseline for follow-up. Management involves margin-controlled surgical excision of invasive disease, with adjunctive cryotherapy and topical Mitomycin C for in situ components. Recurrence occurs in approximately 20% of cases, most commonly within the first 2 years [24,25].

MCC is a rare, aggressive skin tumor that primarily affects individuals over 50 and often arises in sun-exposed areas, especially in immunosuppressed patients. It presents as a rapidly growing, painless red nodule and spreads early via lymphatics, with frequent recurrences within 2–3 years. About 80% of cases are associated with Merkel cell polyomavirus, while others are linked to UV exposure [26]. Diagnosis is based on biopsy and immunohistochemistry (CK20 positive), with staging requiring lymph node assessment and imaging. Treatment involves wide excision and radiotherapy, with additional management for nodal disease. Prognosis is poor (≈50% 5-year survival) [27], though immune checkpoint inhibitors targeting the PD-1/PD-L1 pathway (e.g., pembrolizumab, nivolumab, and avelumab) have improved outcomes in advanced cases [28].

MM of the eyelid is relatively uncommon but represents a potentially life-threatening tumor arising from malignant transformation of melanocytes, typically at the dermo-epidermal junction. Once diagnosed, management should be guided by a specialist multidisciplinary team. Treatment involves surgical excision, typically with ~5 mm margins in the eyelid to balance oncologic control with functional preservation. Sentinel lymph node biopsy is recommended for eyelid tumors with Breslow thickness > 0.8 mm. Advances in targeted therapies and immune checkpoint inhibitors have significantly improved outcomes in advanced disease [29,30].

The management of incompletely excised periocular tumors is a key aspect of margin control. Re-excision is generally recommended due to the risk of residual disease and recurrence. This decision should be guided by histologic subtype, as aggressive variants of BCC (infiltrative, morpheaform, basosquamous) have higher recurrence risk. In more aggressive malignancies, incomplete excision warrants prompt re-excision with wider margins and appropriate staging, often within a multidisciplinary setting [31].

BCC and conjunctival SCC are the most common tumors leading to secondary orbital invasion, reflecting their locally aggressive behavior [32]. The decision to select adjuvant therapies or further surgery should be guided by several factors, including imaging, particularly contrast-enhanced MRI, to accurately define disease extent and inform planning. In cases of orbital extension, a multidisciplinary tumor board should determine the optimal approach, including radiotherapy, Sonic Hedgehog pathway inhibitors, anti–PD-1 therapy, or surgery such as exenteration in selected patients [33].

Imaging for lymph node evaluation is essential in SCC, SGC, MCC, MM, and includes a combination of modalities. MRI is often preferred for local and regional assessment, while ultrasound with color Doppler is used for initial lymph node evaluation and follow-up. Contrast-enhanced CT may be employed for regional and systemic staging when indicated. Sentinel lymph node biopsy provides additional staging information [34].

The main eyelid malignancies and their key clinical and biological characteristics are summarized in Table 1.

### 3.2. Mohs Micrographic Surgery

**Figure 2:**
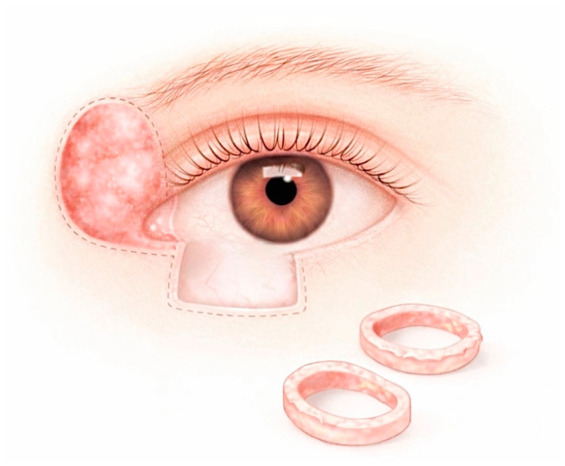
MMS technique. Sequential thin layers of tissue are excised from the base and peripheral margins of the surgical defect and processed as frozen en face sections, allowing comprehensive evaluation of tumor margins and targeted re-excision if residual tumor is detected.

MMS, first developed by Frederic Mohs in the 1940s, is a margin-controlled surgical technique that allows complete circumferential peripheral and deep margin assessment through horizontal (en face) sectioning of excised tissue. This approach enables precise identification of residual tumor and selective re-excision, minimizing unnecessary removal of healthy tissue [35]. During the procedure, the clinically visible tumor is removed together with a thin layer of surrounding tissue. (Figure 2) The specimen is then frozen, mapped, and examined microscopically. If tumor cells are identified at the peripheral or deep margins, additional tissue is selectively excised from the involved area and the process is repeated until complete tumor clearance is achieved [36]. Preoperative histopathological diagnosis is essential to guide intraoperative interpretation, particularly in tumors with ambiguous clinical or histological features [8].

MMS is widely regarded as the reference standard for margin-controlled excision of periocular BCC, especially in tumors with poorly defined borders, aggressive histologic subtypes, or recurrent disease [7]. Its effectiveness is supported by multiple large clinical series. In one of the earliest reports, Mohs described outcomes in over 1400 eyelid BCCs treated with micrographic surgery, demonstrating excellent tumor control with low recurrence rates [35]. Subsequent studies have confirmed these findings, including the Australian Mohs database, which reported a 5-year recurrence rate of approximately 2% for periocular BCC [37], and other large clinical series showing consistently low recurrence rates [38]. A recent systematic review and meta-analysis further supported these results, reporting pooled recurrence rates below 3% [39]. Importantly, consistent with previous reports, a history of prior surgical treatment has been identified as a risk factor for further recurrence following MMS, with recurrent BCCs demonstrating higher recurrence rates compared to primary tumors [38,39].

In addition to BCC, MMS has demonstrated excellent outcomes in periocular SCC. Recent data specifically addressing periocular SCC treated with MMS reported recurrence rates of approximately 6% at a minimum follow-up of 2 years, with low complication rates following oculoplastic reconstruction [40]. These findings are consistent with earlier reports from the Australian Mohs database, which demonstrated recurrence rates as low as ~4% with long-term follow-up [41]. Given the more aggressive biological behavior of SCC compared with BCC, these results further support the role of MMS as a preferred treatment modality in high-risk periocular tumors requiring precise margin control [38].

Beyond keratinocyte carcinomas, MMS has also been investigated in other periocular malignancies [42,43]. In SGC, evidence from a large multicenter cohort study suggests that MMS may offer improved local control compared with wide local excision (WLE), with reported recurrence rates of approximately 15.7% versus 39.6%, respectively, at a median follow-up of 5 years, while metastasis and tumor-related mortality rates appear comparable between the two approaches [44].

Evidence regarding periocular MM remains limited. While MMS, particularly when combined with immunohistochemistry, has demonstrated excellent local control in melanoma in situ [45], its role in invasive melanoma remains controversial. One of the main limitations of MMS in this setting is the inability to reliably assess tumor depth, as en face frozen sections do not allow accurate measurement of Breslow thickness, a key prognostic parameter guiding staging and management [42]. In addition, frozen-section histopathologic analysis may be suboptimal for detecting pagetoid spread and subtle cytologic atypia, both of which are important features in melanoma. For these reasons, MMS is generally not recommended for invasive malignant melanoma, and its use is typically restricted to carefully selected cases of melanoma in situ or lentigo maligna, often in combination with immunohistochemical staining [46].

Despite its advantages, MMS presents several limitations. The procedure may be time-consuming, particularly when multiple stages are required, and its availability is limited by resource and cost considerations. In addition, accurate histopathologic interpretation requires specific expertise; while high interobserver agreement has been reported for BCC diagnosis, agreement for histologic subtyping remains only moderate [8]. Furthermore, frozen section histopathology provides lower tissue quality compared with formalin-fixed paraffin-embedded (FFPE) specimens, potentially affecting both tumor typing and margin assessment. The time-sensitive nature of intraoperative processing may also increase the risk of technical and interpretative errors. For selected cases where immediate reconstruction is not essential, staged excision with paraffin-embedded margin control (slow Mohs) may represent a valuable alternative. This approach allows more accurate histopathologic evaluation, including immunohistochemistry when required, albeit with delayed reconstruction [47]. Morris et al. reported excellent outcomes using slow Mohs, with a 5-year recurrence rate of 0.58%, comparable to or lower than conventional frozen-section MMS, without compromising cosmetic outcomes [48].

### 3.3. Frozen Sections-Controlled Surgical Excision

FSC is a widely used technique for intraoperative histologic margin assessment in oculoplastic surgery. In this approach, the tumor is excised with clinically tumor-free margins, and the surgical specimen is immediately evaluated using frozen section histopathology. When residual tumor is identified at the margins, additional tissue can be removed during the same procedure until tumor-free margins are achieved, thereby allowing immediate reconstruction [9].

**Figure 3:**
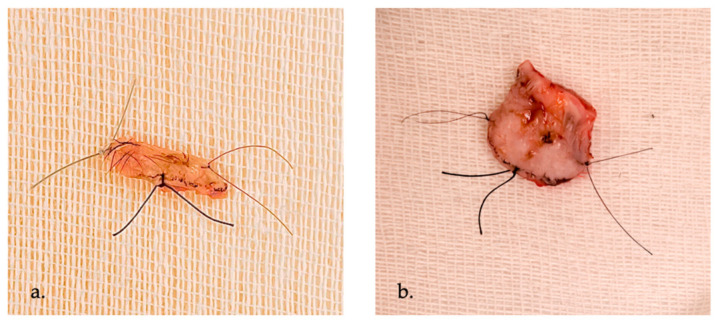
Clinical examples of FSC for BCC. (**a**). Morpheaform BCC of the lower eyelid. (**b**). Ulcerated nodular BCC of the lower eyelid. In both cases, excision was performed with 3 mm clinically tumor-free margins. The surgical specimen is oriented with differently colored sutures marking the medial, lateral, and inferior margins to facilitate intraoperative frozen section margin assessment.

In eyelid tumor surgery, FSC is commonly performed following wedge or pentagonal excision of the lesion. Accurate specimen orientation is essential and is typically achieved by marking the nasal, temporal, and superior or inferior margins with sutures (Figure 3).

**Figure 4:**
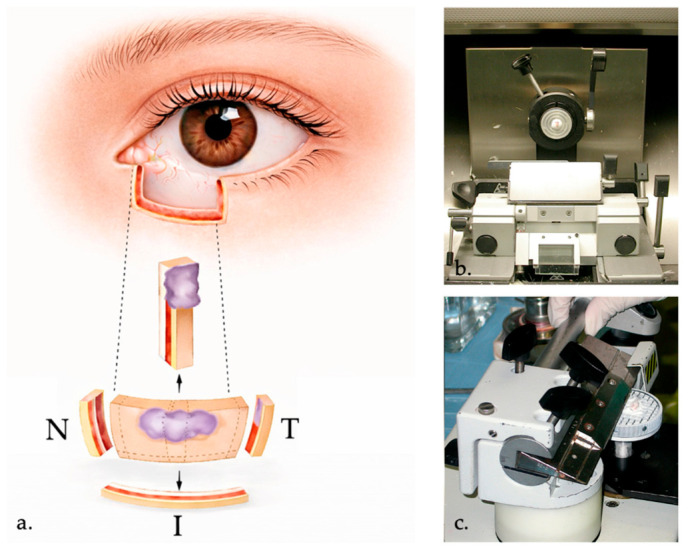
Frozen section processing and instrumentation. (**a**). Schematic representation of specimen orientation: the excised tissue is mapped and the nasal (N), temporal (T), and inferior (I) margins are evaluated using frozen sections. Additional cross-sections may be obtained using a bread-loaf technique to assess the relationship between the tumor and surgical margins. (**b**). Cryostat used for rapid preparation of frozen tissue sections during intraoperative margin assessment. (**c**). Tissue specimen mounted and sectioned within the cryostat using a microtome, illustrating the acquisition of thin sections for immediate histopathologic evaluation.

Margin assessment is performed using frozen sections, most commonly with a bread-loaf technique and multiple-step sections. A central cross-section allows evaluation of the deep margin and confirmation of the histologic diagnosis (Figure 4). All specimens are subsequently processed as permanent paraffin sections to confirm final histopathologic findings [49]. Recommended clinical margins vary depending on tumor type, with at least 2 mm margins for nodular BCC, wider margins of 3 mm for infiltrative BCC and approximately 5 mm for SCC, and margins up to 10 mm for eyelid SGC, MCC, and eyelid MM in accordance with oncologic safety principles [6].

One of the principal advantages of FSC is its practical applicability. The procedure can typically be completed within a single operative session, allowing immediate reconstruction once tumor-free margins are confirmed, thereby reducing overall treatment time and resource utilization [47].

The oncologic effectiveness of FSC has been most extensively studied in periocular BCC. Multiple clinical series have consistently demonstrated low recurrence rates and high rates of intraoperative margin clearance [9,39,50]. In a series of 110 eyelid BCCs treated with FSC and small clinical margins, a recurrence rate of 1.8% was reported after long-term follow-up [9]. Similarly, other authors reported a recurrence rate of 1.4% at a minimum 3-year follow-up, with intraoperative tumor-free margins achieved in 93% of cases [50]. These findings are supported by comparative studies demonstrating that FSC significantly reduces recurrence rates compared with standard excision without margin control [51]. A systematic review and meta-analysis further confirmed these results, reporting pooled recurrence rates of approximately 1.9% for FSC in periocular BCC [39]. As observed with other surgical techniques, recurrent tumors show higher recurrence rates than primary lesions [9].

Evidence regarding the use of FSC in periocular SCC is more limited compared with BCC [52,53]. In a large series of periocular skin carcinomas treated with en face frozen section margin-controlled excision, low recurrence rates were reported, including in SCC, supporting the role of this intraoperative margin assessment in reducing local recurrence risk [53]. Given the more aggressive biological behavior of SCC, including a higher propensity for local invasion and perineural spread, FSC may be particularly useful in selected cases when immediate reconstruction is desired, although robust, technique-specific recurrence data remain less well defined compared with BCC [54].

Similarly, data on SGC are limited, and most studies report outcomes using either WLE with paraffin section control (FPS) or Mohs surgery [38]. FSC may be used as part of a margin-controlled approach, but its role remains less clearly defined, particularly given the potential for pagetoid spread and multifocal disease, which may be difficult to fully assess with frozen section sampling alone [55]. FPS control is a technique in which excised tissue is rapidly processed into paraffin-embedded sections for detailed histopathological evaluation of surgical margins. Compared with FSC, it provides higher-quality and more reliable assessment, but requires more processing time, so reconstruction is usually delayed until clear margins are confirmed. A recent systematic review and meta-analysis found no statistically significant difference in outcomes between WLE and MMS for SGC [4].

Evidence supporting the use of FSC in periocular MM is also limited. Due to the need for precise histopathologic evaluation and, in many cases, immunohistochemical staining, frozen section analysis is generally considered less reliable for melanoma compared with permanent paraffin sections. Consequently, FSC is less commonly employed and is typically restricted to carefully selected cases [30].

In addition to its clinical effectiveness, several technical considerations should be acknowledged. FSC relies on intraoperative histologic evaluation and requires close coordination between the surgeon and pathologist. Because margin assessment is based on representative sampling, complete evaluation of the entire surgical margin may not always be achievable [56]. Furthermore, compared with permanent paraffin-embedded sections, frozen section analysis may provide less detailed histopathologic information in selected cases [47].

The key differences between MMS and FSC are outlined in Table 2, highlighting variations in margin control approach, histologic processing, required expertise, intraoperative workflow, and limitations.

### 3.4. Emerging Imaging Modalities

In recent years, several advanced imaging technologies have been developed to support tumor detection and margin assessment in cutaneous oncology. These techniques aim to complement conventional histopathologic evaluation by enabling rapid, noninvasive, and high-resolution visualization of tumor architecture and boundaries. In particular, imaging modalities capable of identifying subclinical tumor extension have the potential to improve preoperative mapping, optimize margin control, and reduce the number of surgical stages required during procedures such as MMS [57].

Noninvasive imaging techniques—including FCM, RCM, OCT, LC-OCT, and PI—have therefore attracted increasing attention as adjunct tools for real-time tumor assessment [9,10,11,12,13,14]. These modalities provide complementary information at different depths and resolutions, enabling both horizontal and vertical evaluation of skin structures. Although most studies have focused on BCC, these technologies may also assist in the evaluation of other periocular tumors [58].

#### 3.4.1. Fluorescence Confocal Microscopy

FCM is an emerging optical imaging modality that enables rapid, high-resolution visualization of freshly excised tissue without the need for conventional histologic processing [10]. The technique is based on laser scanning confocal microscopy operating in fluorescence mode, typically using acridine orange as a nuclear-specific dye. This fluorescent contrast highlights cell nuclei and allows tumor structures to be distinguished from surrounding stroma with near-histologic resolution comparable to hematoxylin–eosin staining [59]. Large stitched mosaics can be generated from FCM images, providing a panoramic view comparable to Mohs frozen sections while preserving nuclear-level detail. This “digital staining” approach facilitates rapid interpretation and may allow integration into existing Mohs workflows as an alternative to frozen sections [60]. The ability to rapidly acquire high-resolution images within minutes allows for near–real-time analysis of excised tissue and may significantly reduce intraoperative processing time compared with traditional histopathologic techniques [61].

Ex vivo FCM has been extensively investigated for the evaluation of BCC [52,53]. Characteristic morphologic patterns are well described and reproducible across studies. Nodular BCC typically appears as large tumor islands with peripheral palisading, while micronodular variants present as smaller rounded nests. Infiltrative subtypes often appear as thin tumor cords extending within the dermis. Additional diagnostic features include increased nuclear-to-cytoplasmic ratios and characteristic clefting artifacts between tumor islands and surrounding stroma [62]. Several studies have reported high diagnostic accuracy, with sensitivity and specificity values approaching those of conventional frozen section histology [55,56].

FCM has also shown potential in the evaluation of SCC, where tumor silhouettes, keratin pearls, pleomorphic nuclei, and keratinization can be identified. Preliminary studies suggest that FCM may enable rapid intraoperative identification of SCC margins, although diagnostic criteria are less well standardized compared with BCC [63].

Although FCM has demonstrated promising results in cutaneous oncology and MMS, evidence specifically addressing periocular tumors remains limited. Most studies have been conducted on non-periocular skin tumors, and the applicability of FCM for intraoperative margin assessment in eyelid malignancies has not yet been well established. Furthermore, interpretation of FCM images requires specific training and expertise, and standardized diagnostic criteria for periocular tumors are still under development [64].

#### 3.4.2. Reflectance Confocal Microscopy

RCM is a noninvasive imaging technique that provides real-time, in vivo visualization of skin structures at near-cellular resolution using near-infrared laser light. Unlike FCM, RCM does not require exogenous fluorescent dyes, relying instead on intrinsic differences in refractive indices (primarily melanin, keratin, and collagen) to generate image contrast [65].

RCM has demonstrated high diagnostic accuracy in the evaluation of BCC, with reported sensitivity and specificity values of approximately 97% and 89%, respectively. Typical confocal features of BCC include nuclear pleomorphism, elongated basaloid nuclei with nuclear polarization, peripheral palisading, dilated vascular structures, and inflammatory infiltrates surrounding tumor islands. Additional hallmark features include dark silhouettes corresponding to tumor nests and peritumoral clefting, which closely correlate with histopathologic findings [66].

Because RCM can be used directly on the skin surface, it enables noninvasive preoperative tumor mapping and delineation of subclinical lateral tumor extension, which may improve surgical planning. Intraoperative evaluation of wound margins has also been investigated, demonstrating high concordance with histopathologic examination in selected studies [67]. A study evaluated ex vivo RCM for margin assessment in eyelid BCC and reported excellent diagnostic performance, with sensitivity and specificity approaching 100% when compared with conventional histopathology. The authors highlighted the feasibility of rapid intraoperative evaluation of eyelid tumor margins, suggesting that RCM may represent a valuable adjunct for margin-controlled excision in this anatomically complex region. However, the study was limited by a relatively small sample size and the inclusion of a single tumor type, which may limit generalizability [68].

The main limitation of RCM is its relatively shallow penetration depth, typically limited to approximately 200–300 μm. As a result, deeper tumor extensions may not be visualized. Consequently, RCM is generally considered more suitable for evaluating superficial tumor margins rather than assessing deep invasion [59]. Additional limitations include operator dependency, limited field of view, and the need for substantial training to ensure accurate image interpretation [69].

#### 3.4.3. Optical Coherence Tomography

OCT is a noninvasive imaging modality that uses low-coherence infrared light to generate real-time, cross-sectional and en face images of tissue microarchitecture [70]. By measuring the backscattering of light from different skin layers, OCT enables visualization of the epidermis, dermoepidermal junction, and superficial dermis, with a penetration depth typically ranging from 1 to 1.5 mm and spatial resolution in the micrometer range [71]. These characteristics make OCT particularly suitable for evaluating tumor morphology and delineating subclinical extension in cutaneous malignancies [72,73,74,75].

OCT has been increasingly investigated for periocular tumors. In a prospective study of 58 eyelid and periocular lesions, OCT successfully differentiated malignant from benign tumors [12]. In BCC, several characteristic OCT features have been described and correlated with histopathologic findings, including hyporeflective dermal nodules, disruption of the dermoepidermal junction, and lobulated tumor nests [74]. Furthermore, nodular BCC typically appears as well-defined hyporeflective dermal islands, whereas infiltrative and morpheaform subtypes may present as smaller, irregular aggregates or grape-like clusters extending into the dermis, but lower diagnostic accuracy is reported [75].

OCT has also been investigated for preoperative tumor mapping and intraoperative margin assessment [76,77,78,79,80]. In a prospective study evaluating biopsy-proven BCC prior to MMS, OCT demonstrated an overall diagnostic accuracy of approximately 95.5% compared with histopathology. Preoperative OCT successfully predicted the need for additional surgical stages in most cases and correctly identified tumor absence in lesions that had regressed following diagnostic biopsy [79]. Similarly, Ex vivo OCT applications during Mohs surgery have also demonstrated promising results. In a prospective study of 73 facial BCCs, OCT achieved a sensitivity of 81% and a specificity of 94% for identifying tumor-positive margins, with an overall diagnostic accuracy of 93% [80]. These findings suggest that OCT may reduce the number of Mohs stages and help optimize surgical planning by improving delineation of tumor margins. However, OCT performance appears to be subtype-dependent, with lower accuracy in aggressive variants such as infiltrative or morpheaform BCC and potential underestimation of peripheral tumor extension when discrete sampling protocols are used, highlighting the need for cautious interpretation and complementary imaging approaches [79].

Limitations also extend to other periocular malignancies. For SCC, current evidence remains limited, and larger case series are required to better define characteristic OCT features and diagnostic criteria. Similarly, findings in SGC remain heterogeneous, with reported features including epithelial thickening and hyperreflective nodules or halos [70]. Additional limitations include the need for operator experience, potential motion artifacts related to eyelid curvature, and limited penetration depth for evaluating deeply infiltrative tumors. Furthermore, most available studies involve relatively small patient cohorts, and standardized protocols for intraoperative margin assessment in periocular tumors remain under development [81].

#### 3.4.4. Combined RCM-OCT

A new, promising imaging modality utilizes a single probe to combine RCM with OCT [82]. This enables real-time, 3-dimensional analysis of the tumor with simultaneous display of the cross-sectional OCT and en face RCM images [57]. This multimodal approach has been investigated primarily in BCC, where combined RCM–OCT imaging provides complementary structural and cellular information. The integration of these modalities improves detection of irregular epidermal architecture, tumor margins, and subclinical extension, thereby enhancing diagnostic confidence and presurgical planning [83].

In a prospective study of 38 BCCs scheduled for Mohs surgery, combined RCM–OCT imaging demonstrated high diagnostic performance, with overall agreement of 91.1% compared with frozen section histology. Sensitivity and specificity were 82.6% and 93.8%, respectively, and OCT depth measurements strongly correlated with histologic tumor depth, supporting its role in presurgical margin mapping [82]. However, current evidence remains limited to cutaneous lesions outside the periocular region, and no studies to date have specifically evaluated the intraoperative use of combined RCM–OCT imaging for margin assessment in eyelid tumors, highlighting an important area for future investigation.

#### 3.4.5. Line-Field Confocal OCT

LC-OCT is an emerging non-invasive imaging modality that integrates OCT with RCM using line-field illumination and detection. This technology effectively bridges the gap between RCM and OCT by merging the high cellular resolution of the former (~1 µm) with the superior penetration depth of the latter (~500 µm) [84]. LC-OCT enables real-time, high-definition visualization of skin architecture in vertical, horizontal, and three-dimensional modes, allowing detailed assessment of epidermal and superficial dermal structures with near-histologic resolution [85]. Systematic reviews have positioned LC-OCT among the highest-performing non-invasive imaging techniques for BCC assessment when compared with established modalities [86]. More recent studies have further demonstrated that LC-OCT, particularly when combined with AI, can significantly improve diagnostic accuracy and consistency across clinicians with varying levels of expertise [87,88].

In the periocular region, LC-OCT has shown promising diagnostic performance. In a study of 51 eyelid lesions, a diagnostic concordance of 92.1% was reported between LC-OCT findings and histopathology. Characteristic morphological features included dermal lobules, peripheral clefting, and altered collagen bundles in BCC; architectural disorganization, hyperkeratosis, and atypical keratinocytes in SCC; and atypical dendritic or roundish cells with pagetoid spread in MM. These findings highlight the potential of LC-OCT for non-invasive characterization of periocular tumors [13].

**Figure 5:**
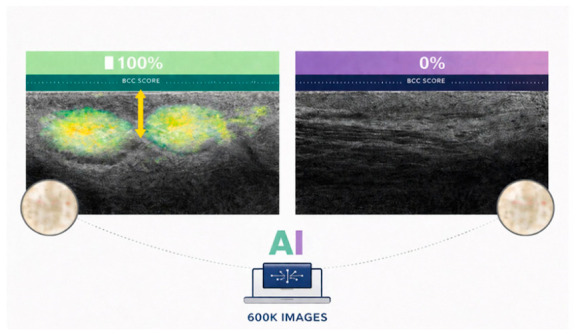
Example of AI–assisted LC-OCT analysis for BCC detection. AI-generated probability maps are superimposed on LC-OCT images, highlighting areas suspicious for BCC with color-coded heatmaps. The left panel shows a lesion with a high AI-derived BCC score (100%), with highlighted regions corresponding to tumor lobules, whereas the right panel demonstrates normal skin with a low BCC score (0%) and absence of suspicious features.

Beyond diagnostic applications, LC-OCT has also been investigated for perioperative assessment of cutaneous tumors and for monitoring tumor clearance after local therapies [89,90,91]. By enabling real-time visualization of tumor architecture, LC-OCT allows clinicians to delineate lesion margins more accurately before surgery, potentially improving surgical planning and minimizing the removal of healthy tissue [92]. In a case–control study including 63 high-risk facial BCCs, preoperative LC-OCT margin mapping significantly reduced the number of MMS stages compared with clinical and dermoscopic assessment alone (mean 1.23 vs. 1.89 stages; *p* = 0.007), demonstrating improved tumor delineation and more efficient surgical management [90]. In a study including 50 cutaneous lesions, AI-assisted LC-OCT co-localized with wide-field dermoscopy was evaluated for lateral margin mapping both in vivo and ex vivo. A promising correlation between imaging and histological results was observed, supporting the technical reliability of this approach. Illustrative cases from this series demonstrated that LC-OCT could accurately predict both complete tumor excision and residual tumor involvement, with concordant findings between in vivo assessment, ex vivo imaging of the excised specimen, and subsequent histopathological analysis (Figure 5) [88]. Practical limitations of ex vivo LC-OCT must be considered, as uneven specimen surfaces, tissue folding, and air bubbles at the tissue–glass interface may impair optical coupling and hinder visualization of deeper margins [91].

#### 3.4.6. Photoacoustic Imaging

PI is a hybrid modality that combines laser light with ultrasound, exploiting the differential absorption of optical energy by endogenous tissue chromophores. Light pulses induce thermoelastic expansion, which generates acoustic waves subsequently detected by an ultrasound transducer, enabling high-resolution three-dimensional visualization of the molecular composition of tissues. Compared to purely optical methods, PI achieves greater penetration depth while maintaining molecular specificity [14,71].

Its application to margin assessment during excision of periocular skin tumors has recently attracted growing interest [14,93]. In an ex vivo study of malignant melanoma, BCC, and SCC of the eyelid and periocular region, PI revealed distinct spectral signatures for each tumor type. BCCs showed predominant contributions from oxygenated and deoxygenated hemoglobin, suggestive of tumor angiogenesis, whereas SCCs were characterized by increased melanin absorption and decreased collagen signals. Malignant melanoma spectra were reconstructed almost entirely by melanin absorption. The difference between BCC and adjacent healthy tissue was statistically significant across the full spectral range of 680–970 nm, with strong spectral fitting [94].

A case report further demonstrated the ability of PI to detect residual tumor at the medial margin following pentagonal excision of an eyelid BCC. This finding was later confirmed histologically, underscoring the potential of PI for intraoperative margin control and reduction in re-excisions [14].

Additional work has established the feasibility of distinguishing normal eyelid structures such as skin, orbicularis oculi muscle, and tarsal plate based on their specific spectral profiles, which is a critical prerequisite for clinical application during eyelid tumor surgery [95]. However, current evidence remains limited to small, predominantly ex vivo studies, and technical constraints—including dependence on endogenous chromophores, reduced resolution at greater depths, and limited device availability—currently restrict the routine intraoperative use of photoacoustic imaging for eyelid tumor margin assessment [14,96].

A comparative overview of emerging imaging modalities for intraoperative margin assessment is provided in Table 3.

### 3.5. Artificial Intelligence–Based Approaches

AI has emerged as a promising tool to support intraoperative pathology and improve margin assessment in cutaneous oncology [97]. A recent systematic review identified 18 studies employing machine learning models—most commonly convolutional neural networks—for analysis of frozen section images during MMS. These models demonstrated strong diagnostic performance, with reported area under the receiver operating characteristic curve (AUC) values up to 0.997 for BCC detection, in some cases matching or exceeding the accuracy of board-certified Mohs surgeons. Importantly, physician performance improved when combined with AI outputs, highlighting the potential of collaborative human–AI decision-making [98].

More broadly, AI applications in MMS have been investigated for tumor identification, margin assessment, stage prediction, and workflow optimization. A recent systematic review and meta-analysis reported consistently high diagnostic performance and improved efficiency across these applications, suggesting that AI may enhance both accuracy and operative workflow in margin-controlled surgery [99].

AI has also been applied to emerging optical imaging technologies. In a feasibility study using high-resolution full-field OCT on 113 excised tissues, expert readers achieved a sensitivity of 93.7% but relatively low specificity (58.3%). AI-assisted analysis improved specificity to 81.2%, highlighting the potential of machine learning to enhance interpretation of optical imaging modalities and facilitate intraoperative decision-making [72].

Despite these promising developments, current AI applications remain limited by small training datasets, variability in imaging protocols, and the need for external validation across institutions [100].

## 4. Conclusions

Eyelid malignancies present unique surgical challenges due to the anatomical complexity and functional importance of the periocular region. Achieving complete tumor removal while preserving eyelid function and cosmesis remains the primary objective of surgical management. Intraoperative margin control plays a critical role in ensuring oncologic safety while minimizing unnecessary tissue sacrifice.

MMS and FSC are both associated with very low and comparable recurrence rates in periocular BCC, with no significant difference in safety demonstrated across studies. From a practical clinical perspective, the choice between MMS and FSC is guided by tumor-related and patient-specific factors rather than a single standardized algorithm. MMS provides comprehensive circumferential margin assessment through en face sectioning and remains widely regarded as the reference standard for tumors with poorly defined margins, aggressive histologic subtypes, or recurrent disease, and those located in anatomically critical areas such as the medial canthus, where subclinical spread is common [6,34]. However, FSC continues to represent a reliable and widely used alternative in oculoplastic practice. When performed with accurate specimen orientation and careful histopathologic evaluation, FSC can achieve recurrence rates comparable to those reported for MMS while allowing single-stage excision and reconstruction [37,38,39]. From a cost perspective, MMS is more resource-intensive and time-consuming due to repeated intraoperative margin assessment, and is therefore generally more expensive. FSC is typically more accessible and likely less costly, with current evidence not showing a clear cost-effectiveness advantage for MMS [101].

Recent technological advances are expanding the role of imaging in tumor detection and margin assessment beyond conventional histopathology. Noninvasive imaging modalities —including FCM, RCM, OCT, LC-OCT, and PI—have shown promising capabilities in visualizing tumor architecture and identifying subclinical extension. These technologies may support presurgical mapping, guide intraoperative decision-making, and potentially reduce operative time and the number of surgical stages required [12,14,58,62,68,90].

AI–assisted analysis is also emerging as a valuable adjunct for interpreting histologic and imaging data. Machine learning algorithms have demonstrated high diagnostic accuracy in detecting BCC and other cutaneous malignancies, with the potential to enhance efficiency and reproducibility in margin assessment. However, most available data derive from early clinical or experimental studies, and further validation is required before widespread clinical adoption. Consequently, histopathologic evaluation remains the cornerstone of intraoperative margin control in eyelid tumor surgery [99,100].

Among emerging modalities, in vivo LC-OCT–based margin assessment appears particularly promising for improving surgical efficiency and reducing healthcare resource utilization [13]. More accurate preoperative delineation of tumor boundaries may limit unnecessary tissue removal, an important consideration in the cosmetically and functionally sensitive periocular region. Improved margin definition may also decrease the number of surgical stages, particularly in micrographic procedures, thereby reducing operative time, histopathologic workload, and personnel demands. Furthermore, AI-supported digital workflows may enhance documentation, reproducibility, and integration into routine clinical practice without requiring substantial additional infrastructure [88,90].

Future research should focus on validating these advanced imaging technologies and AI-driven approaches in larger periocular cohorts, with the goal of improving surgical precision, optimizing workflow efficiency, and ultimately enhancing patient outcomes.

## Figures and Tables

**Table 1:** Overview of common eyelid malignancies and their characteristics.

Tumor Type	Epidemiology	Cell of Origin	Typical Clinical Features	Biological Behavior
**Basal cell carcinoma**	~90% of eyelid malignancies	Basal keratinocytes of the epidermis	Pearly papule with telangiectasia, possible ulceration; may cause eyelash loss when involving the eyelid margin	Locally invasive with low metastatic potential; may show subclinical extension [16]
**Squamous cell carcinoma**	~5–10%	Keratinocytes of the epidermis	Hyperkeratotic plaque or ulcerated lesion; may arise from actinic keratosis	More aggressive than BCC; potential for PNI and regional metastasis [19,20,21]
**Sebaceous gland carcinoma**	<5%	Sebaceous glands (mainly Meibomian glands)	Yellowish or nodular lesion, often mimicking chalazion; may show pagetoid spread along conjunctiva	Aggressive tumor with risk of local recurrence and regional or distant metastasis [23]
**Merkel cell carcinoma**	Rare	Neuroendocrine cells of the skin	Rapidly growing, painless reddish-violet nodule on sun-exposed skin	Highly aggressive tumor with early lymphatic spread and poor prognosis [26,27]
**Malignant melanoma**	Rare	Melanocytes at the dermo-epidermal junction	Pigmented lesion, irregular borders; may arise from nevus or lentigo maligna	Potentially life-threatening with high metastatic potential; management guided by Breslow thickness [29,30]

**Table 2:** Comparison between MMS and FSC.

Feature	Mohs Micrographic Surgery (MMS)	Frozen Section–Controlled Excision (FSC)
**Margin assessment**	Complete circumferential peripheral and deep margin control (en face sections)	Sampling-based margin assessment (vertical bread-loaf sections or en face depending on technique)
**Tissue preservation**	Maximal tissue preservation	Good tissue preservation, but less precise than MMS
**Reconstruction**	Often delayed until margin clearance; sometimes staged	Performed during the same surgical session
**Number of surgical stages**	Multiple stages often required	Single-stage excision with possible additional resections
**Recurrence rates**	Very low (≈1–3% for periocular BCC)	Low (≈1–2% in reported periocular series)
**Histologic processing**	Frozen sections with en face mapping; no formalin-fixed paraffin-embedded (FFPE) tissue	Intraoperative frozen sections + postoperative FFPE sections for final diagnosis and quality assurance
**Anesthesia**	Usually local anesthesia in an outpatient dermatologic setting	Local anesthesia with sedation or general anesthesia depending on the surgical context
**Availability**	Limited to specialized centers	More widely available
**Operative time**	Longer, due to multiple stages	Shorter
**Cost**	Higher overall cost	Lower cost compared with MMS
**Technical expertise required**	Mohs-trained dermatologist performing both surgery and histologic interpretation	Surgeon (often oculoplastics-trained) with intraoperative support from a pathologist
**Accuracy of margin control**	Highest accuracy	High accuracy but sampling-dependent
**Indications**	High-risk tumors, recurrent lesions, aggressive histologic subtypes, medial canthus tumors	Primary tumors, well-defined lesions, centers without MMS availability
**Limitations**	Time-consuming; resource-intensive; limited availability; lower histologic detail due to frozen sections; not recommended for invasive malignant melanoma	Sampling error; lower histologic detail due to frozen sections (although FFPE improves final assessment)
**Alternative approach**	Slow Mohs (paraffin-embedded sections)	Fast paraffin section control and delayed reconstruction

**Table 3:** Comparison of Emerging Imaging Modalities for Intraoperative Margin Assessment in Eyelid Tumor Surgery.

Imaging Modality	Principle	Resolution/Penetration	Main Applications	Advantages	Limitations	Evidence in Periocular Tumors
**Fluorescence Confocal Microscopy (FCM)**	Laser scanning confocal microscopy with fluorescent dye (e.g., acridine orange)	Near-histologic resolution; ex vivo imaging	Intraoperative margin assessment, Mohs surgery adjunct	Rapid imaging, digital staining, large mosaics, high diagnostic accuracy for BCC	Requires tissue excision, training required, limited periocular data	Limited evidence; promising for eyelid BCC margin assessment [55,56,57,58,59,60]
**Reflectance Confocal Microscopy (RCM)**	Near-infrared laser; reflectance from intrinsic tissue structures	~200–300 µm penetration; cellular resolution	Noninvasive tumor mapping, superficial margin evaluation	Real-time in vivo imaging, high sensitivity	Limited depth, operator dependent, small field of view	Limited but promising for preoperative mapping [61,62,63,64,65]
**Optical Coherence Tomography (OCT)**	Low-coherence infrared light; cross-sectional imaging	1–1.5 mm penetration; micrometer resolution	Tumor characterization, margin delineation, presurgical mapping	Noninvasive, deeper penetration than RCM, real-time imaging	Lower accuracy in infiltrative subtypes, motion artifacts, operator dependence	Increasing periocular evidence; diagnostic accuracy ~95% in BCC [11,66,67,68,69,70,71,72,73,74,75,76]
**Combined RCM–OCT**	Single probe combining RCM (en face) and OCT (cross-sectional)	High resolution + moderate penetration	3D tumor mapping, presurgical planning	Complementary structural information, improved diagnostic performance	Limited availability, minimal periocular-specific studies	No dedicated eyelid tumor studies yet [78,79]
**Line-field Confocal OCT (LC-OCT)**	Combines confocal microscopy with OCT using line-field illumination	~1 µm resolution; ~500 µm penetration	Tumor diagnosis, margin mapping, therapy monitoring	Near-histologic resolution, 3D imaging, AI integration possible	Optical artifacts in ex vivo imaging, limited penetration depth	Strong periocular data; ~92% concordance with histopathology [12,80,81,82,83,84,85,86,87,88]
**Photoacoustic Imaging (PI)**	Laser-induced ultrasound based on chromophore absorption	Deeper penetration with molecular contrast	Tumor detection, intraoperative margin assessment	Molecular specificity, 3D imaging, differentiates tumor types	Limited availability, mostly ex vivo data, lower resolution at depth	Early evidence in eyelid tumors; promising but preliminary [13,89,90,91,92]

## Data Availability

No new data were created or analyzed in this study. Data sharing is not applicable to this article.
